# Nutrient-doped synthetic silicates for enhanced weathering, remineralization and fertilization on agricultural lands of global cold regions – A perspective on the research ahead

**DOI:** 10.1016/j.isci.2022.105556

**Published:** 2022-11-13

**Authors:** Andrea Hicks, Pratik Dholabhai, Asif Ali, Rafael M. Santos

**Affiliations:** 1 Department of Civil and Environmental Engineering, University of Wisconsin-Madison, Madison, WI, USA; 2 School of Physics and Astronomy, Rochester Institute of Technology, Rochester, NY, USA; 3 School of Engineering, University of Guelph, Guelph, ON, Canada

**Keywords:** Agricultural science, Relation between agriculture and environment, Materials science, Materials chemistry

## Abstract

There is now a dire demand for negative emissions technologies (which sequester CO_2_ from the atmosphere) that can be rapidly deployed, are scalable, and are demonstrably safe and effective. Enhanced weathering of silicate minerals has demonstrated a significant potential for CO_2_ capture and sequestration by the formation of pedogenic carbonates in soils, subsoils, and sediments. This technique has also been shown to deliver fruitful results in terms of improving soil health, and in turn plant health, through remineralization. The silicate minerals that possess the highest weathering rates (e.g., wollastonite), are relatively rare in nature, whereas the abundant ones (e.g., anorthite and forsterite) have a slower pace of weathering, especially in colder and drier climates such as found in the extensive agricultural lands of Western Canada and the Western United States. Herein, we offer a perspective on the opportunities for computational studies targeting atomic-scale interaction of CO_2_ with silicates and synthesis of fast-weathering silicates (such as larnite and bredigite), whose composition can be tuned to also support soil fertilization and remineralization, and whose production must be integrated with green and carbon-neutral technologies to ensure net-negative life cycle emissions.

## Graphical abstract

**Figure:**
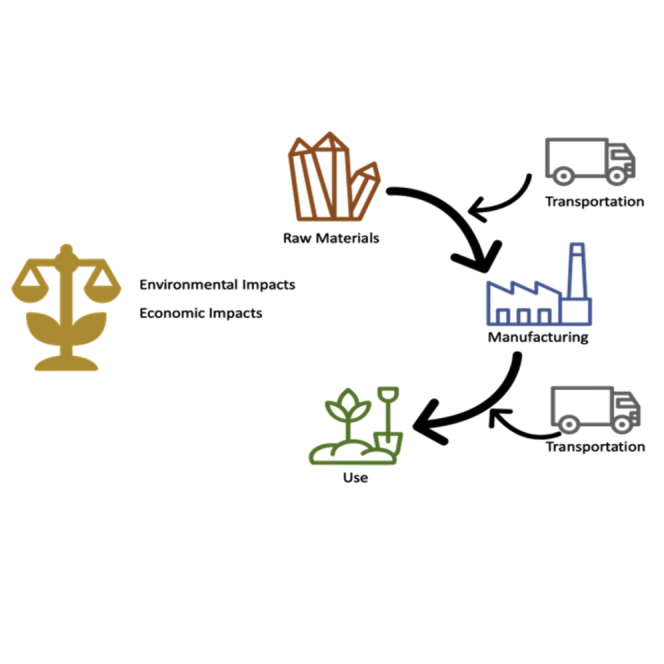

## Background and motivation

**Figure 1:**
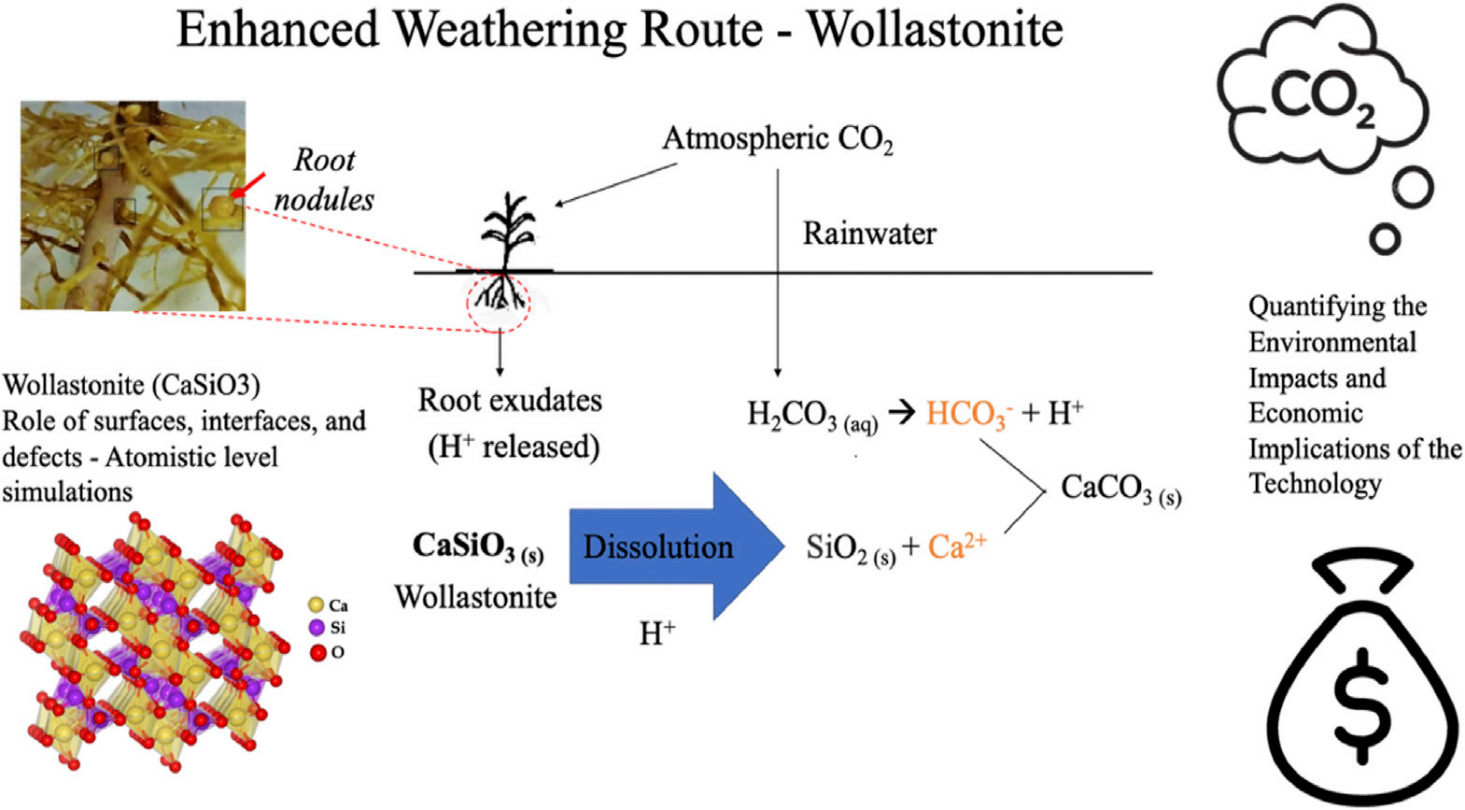
Overview of the potential for sustainable ERW

With no single carbon capture and sequestration solution able to limit the global temperature rise to 1.5–2°C by 2100, additional negative emissions technologies, for carbon drawdown, are required to complement the current mitigation approaches. Enhanced rock weathering (ERW) is one negative emissions technology that, applied globally, could remove gigatonnes of CO_2_ per year from the atmosphere.^1^^,^^2^ In ERW, CO_2_ removal occurs through the exposure of certain silicate minerals to the atmosphere, soil pore water, and oceanic waters, whereby CO_2_ is transiently captured as soluble bicarbonates and permanently trapped as thermodynamically stable carbonates.^1^^,^^2^ ERW applications were originally proposed for agricultural and forestlands and coastal areas, where water, air, and (micro)biological activities could work together to weather minerals and transport sequestered CO_2_ to long-term carbon sinks.^3^ The inorganic carbon capacity of soils is enhanced by amending them with calcium- or magnesium-rich silicate rocks,^4^^,^^5^ which leads to enhanced weathering and in turn accelerates CO_2_ sequestration via mineral carbonation (Figure 1). Soil inorganic carbon produced by ERW near the soil surface gradually migrates to the subsoil, becoming part of the natural pedogenic carbonate pool.^6^ Farms provide an excellent setting for large-scale ERW through mineral soil amendments,^7^ because of the available infrastructure and experience with spreading comminuted mineral amendments (e.g., liming and phosphate rock), and the typically suitable soil properties and local climate: sufficient soil acidity and rainfall are key drivers of silicate weathering.

**Figure 2:**
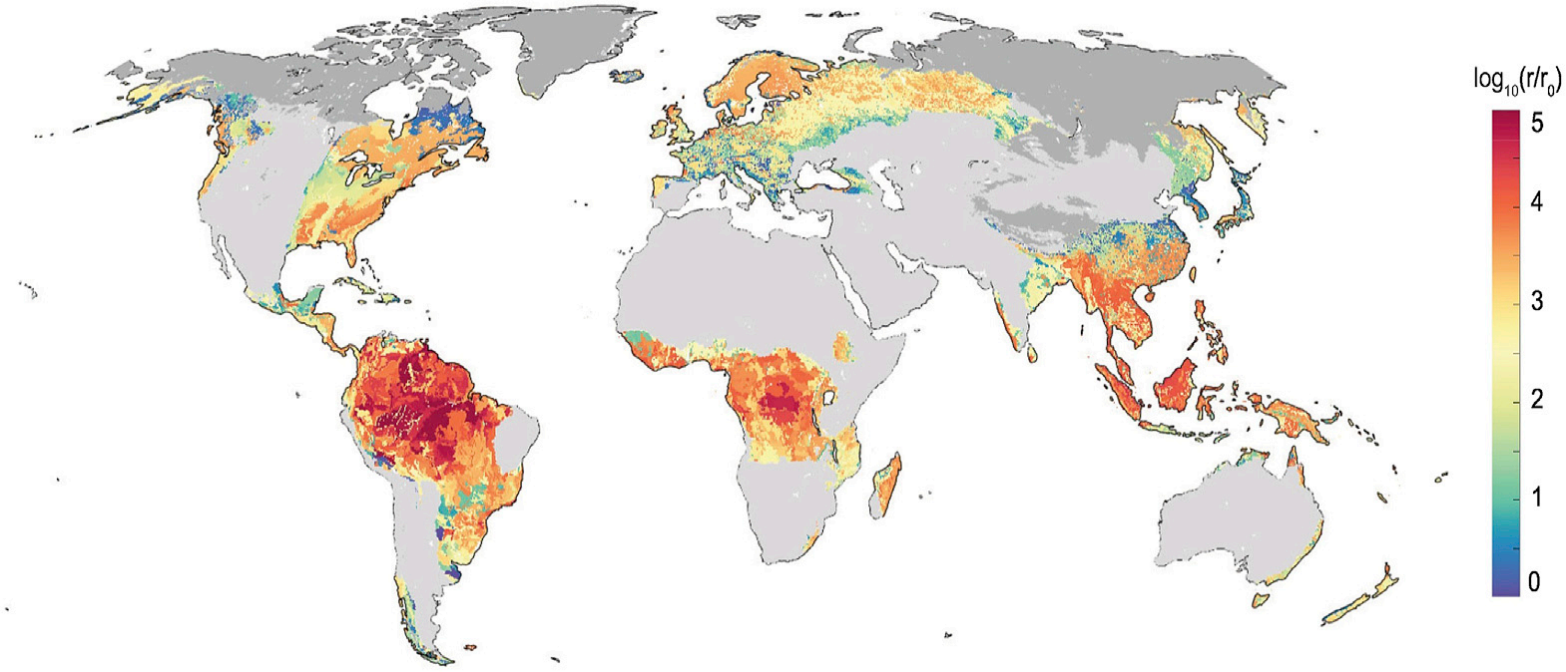
Alkalinization carbon capture efficiency in the world’s topsoils (0–30 cm) Light and dark gray regions are either too arid (aridity index above 3) or too cold (average temperature below 0°C) for ERW application.^10^ Re-used with permission from Elsevier (5417940776303).

Recent ERW research has focused on tropical and temperate regions of the world owing to the relatively slow weathering rates of the most abundant silicates (basalt and olivine) in the colder and dryer regions of the world, wherein soils also tend to be more basic because of slower weathering of pre-existing soil carbonates.^8^ For example, Canada possesses extensive agricultural lands, but the conditions of much of it, especially in the Prairies, for ERW of basalt and olivine are not suitable, because it may take centuries to capture any notable CO_2_ from the environment. In a recent investigation by Strandmann et al.,^9^ the dissolution of olivine added to soil at 4°C was reported to be orders of magnitude slower than under optimal conditions for ERW. Another recent investigation by Bertagni et al.^10^ plotted the global mineral dissolution rates, as shown in Figure 2. According to this plot, major portions of the world, such as much of Western Canada and the Western United States, among several other countries, will not be able to gain benefit from the ERW technique, owing to climatic limitations. Of Canada’s 62,195,226 hectares of farming land, 39% is in the province of Saskatchewan, and 32% is in the province of Alberta^11^; the agricultural lands in these two provinces have substantially colder and drier climates, and consequently more alkaline soils^12^ than those in the provinces of Ontario and Quebec. Similarly in the US, the northern plains (North Dakota, South Dakota, Nebraska, and Kansas) account for the largest share of cropland at 39.2 million hectares.^13^ The colder climates with lower rainfall rates and higher pH of soil make it difficult to weather naturally occurring silicate minerals. This research gap needs to be addressed to tap the ERW potential more broadly to tackle climate change.

Although wollastonite (CaSiO_3_) has been demonstrated to weather relatively fast (in the order of a crop season) in suitable agricultural soil,^7^ the present challenge is that such reactive naturally occurring silicates are less abundant, and global stocks in the order of hundreds of megatonnes^14^ would rapidly deplete once ERW scales up. Moreover, to maximize net-negative emissions, silicates should be available near point-of-use; yet, these scarce silicates are located in sparse ore reserves. For example, the currently commercialized wollastonite reserves in Canada are located in Ontario and Quebec, thus far from Alberta or Saskatchewan. The transport of minerals has been shown to be the largest factor affecting net-carbon sequestration potential.^15^ This perspective introduces the idea of synthetic silicates and their potential use in ERW in colder, dryer, and remote locations. The idea is that synthetic minerals can be made to be significantly more reactive than their natural counterparts,^16^ and their composition can be tailored to provide additional agronomic benefits.^17^^,^^18^ Also, the silicate production process can potentially be integrated with other technologies (geological sequestration and cement production),^19^^,^^20^ to minimize the overall environmental footprint.

Renforth^21^ recently presented the idea of synthesizing ikaite (CaCO_3_·6H_2_O) as a fast-weathering mineral that can be used in cold seawater for CO_2_ capture. The synthesis of this mineral is not novel, as it is based on established geochemical processes, however, its production (which includes pumping/compressing/cooling operations) can be achieved in a CO_2_-negative manner if very low-carbon footprint electricity is used. Likewise, the methodology and conditions for the synthesis of silicate minerals, via solid-state sintering, are well established,^16^ but there is substantial research scope in tuning their composition of mineralogical properties for ERW in colder regions. In addition, new developments in electricity-driven metallurgical and cement-making processes open new opportunities for industrial synergism and symbiosis. The increase in climate change and CO_2_ emissions awareness is shifting the global community’s attention toward clean energy conversion and renewable energy resources,^22^^,^^23^ and as such these industries are also starting to understand the importance of switching towards green equipment and energy resources. For example, an Austrian steelmaker is converting three of its blast furnace plants to electric arc furnaces,^24^ and a Canadian steelmaker in Ontario is on the same path.^25^ The adoption of such environmentally friendly production systems can make the adoption of synthetic silicate minerals for ERW viable. Another attractive opportunity is the integration of silicate production processes with technologies such as cement production and geological sequestration,^19^^,^^20^ which would allow for minimizing the environmental footprint from cradle (silicate synthesis) to grave (soil amendment).

**Figure 3:**
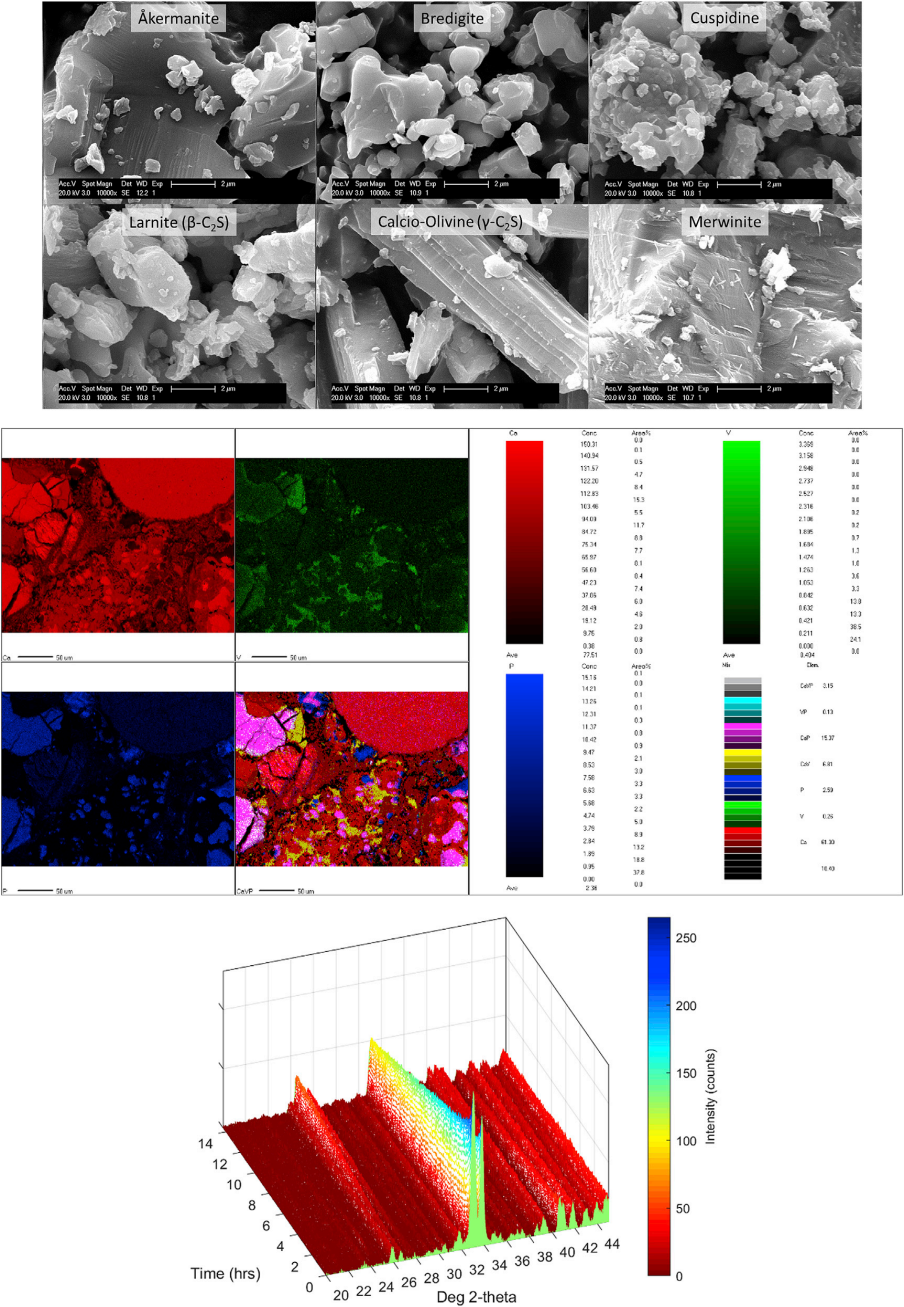
Synthetic and slag-occurring silicates and their weathering/carbonation reactivity Top: Morphology of fresh synthetic mineral particles (unpublished data from work reported in Bodor et al.^16^), inspected by scanning electron microscopy (SEM) (scale bar 2 μm). Middle: Distribution of elemental components in a polished section of a carbonated basic oxygen furnace (BOF) slag <0.5 mm sample embedded in resin, obtained by electron probe micro-analysis (EPMA)^28^ (scale bar 50 μm); re-used with permission from Elsevier (5171571410891). Bottom: 3D high-pressure X-ray diffraction (HXRD) diffractogram of synthetic bredigite carbonated in wet CO_2_ at 70 °C and 6 bar^27^; re-used with permission from Elsevier (5171580487674).

The research hypothesis for synthesizing reactive silicate minerals for ERW application is that these materials can be produced with higher weathering rates than their natural counterparts. Fast-weathering silicates such as merwinite and bredigite are found in steelmaking slags (Figure 3), and these materials have been shown to be more reactive than natural minerals such as olivine both under chemoorganotrophic bioleaching conditions^26^ and under accelerated carbonation conditions.^27^ It has also been found that the composition of synthetic silicates can be customized,^16^ with the potential to harness enhanced benefits in agricultural yields by doping silicate minerals with plant nutrients, much like P and V are sequestered in some metallurgical slags (Figure 3) during steel refining.^28^

The following research objectives and hypotheses should be investigated to evaluate the technical and practical feasibility of utilizing synthetic silicates for ERW in agricultural settings:

• Synthesizing various fast-weathering silicate minerals via solid-state sintering. The hypothesis is that those silicate minerals with a high Ca:Si ratio, and not containing Al, will weather significantly faster and more predictably in soil ERW settings. Such minerals include (Figure 3): åkermanite (Ca_2_MgSi_2_O_7_), bredigite (Ca_7_Mg(SiO_4_)_4_), cuspidine (Ca_4_Si_2_O_7_F_2_), larnite (β-Ca_2_SiO_4_), calcio-olivine (γ-Ca_2_SiO_4_), merwinite (Ca_3_Mg(SiO_4_)_2_), and pseudowollastonite (β-CaSiO_3_).
• Investigating the doping of plant nutrients (e.g., P, K, Mo, and Se) into the silicates, to increase their fertilizing ability. The hypothesis is that as the silicates weather, they will release plant nutrients, with amorphous silica,^18^ and this can be an effective way of fertilizing and remineralizing soil while sequestering carbon.
• Investigating mechanical activation and degree of crystallinity as means to increase the weathering rate of synthetic silicates. The hypothesis is that silicates with crystal defects and disorders in their crystal structure will be more reactive under ERW soil settings.
• Testing the synthesized materials in soils to determine their fertilizing ability and CO_2_ sequestration ability. The hypothesis is that the synthetic silicates will behave similarly to how steelmaking slags are known to rapidly weather and carbonate but will be safer because of the tuned chemical composition, and their behavior will be more predictable because of controlled mineralogy.
• Conceptualizing an integrated industrial process to manufacture these synthetic silicates in a net-negative carbon way. The hypothesis is that synthetic silicate production can be combined with carbon capture and sequestration (CCS) via geological carbon storage, to enable supplying free alkaline earth metal oxides from natural carbonates, in a similar approach to that used in the production of carbon-neutral cement via integrated calcination-CCS.^29^

## Potential field trials

A utilitarian role in the global carbon cycle is played by natural soils, as it demonstrates an approximate reservoir of 2500 Gt of carbon € that far exceeds the atmospheric C reservoir of 800 GtC.^30^^,^^31^ There are two major natural pathways through which the atmospheric CO_2_ is regulated by pedogenic processes in soils: by photosynthesis, which influences the soil organic carbon (SOC) pool, and via weathering of alkaline minerals, which controls the soil inorganic carbon (SIC) pool. Although many investigations to date have been conducted for increasing the SOC pool capacity,^32^^,^^33^^,^^34^^,^^35^ the SIC pool has received relatively limited attention, especially the amendment of soils by alkaline silicate minerals for building up the capacity of the inorganic pathway.

Given that the CO_2_ sequestration capacity of alkaline minerals accessible deposits outnumbers the estimates of carbon content present in fossil fuel reserves,^1^ some of these minerals can play a significant role in minimizing the hazardous effects of excessive CO_2_ presence in the atmosphere. The ERW approach has candidate materials made up of calcium and magnesium silicates, including the serpentine group ((Mg,Fe^II^)_3_Si_2_O_5_(OH)_4_) minerals, the olivine group ((Mg,Fe)_2_SiO_4_) minerals such as fayalite (Fe_2_SiO_4_) and forsterite (Mg_2_SiO_4_), and the pyroxene group ((Ca,Na,Fe^II^,Mg)(Cr,Al,Fe^III^,Mg,Mn,Ti,V)Si_2_O_6_) minerals such as enstatite (MgSiO_3_) and wollastonite (CaSiO_3_).^36^ As an example, all carbon that can be expected to be emitted from the currently recognized reserves of natural gas, oil, and coal can in theory be completely sequestered by the global reserves of serpentine and peridotite (olivine and pyroxene).^37^

The process of ERW consists of the amendment of soils with alkaline earth minerals.^38^ Magnesium- or calcium-rich silicate minerals are finely milled/crushed, and the powder is added to soils. The mineral weathering process releases the alkaline earth metal cations. Concurrently, the carbonate anions originating from the soil microbial process or the atmosphere, are dissolved in the soil pore water. Alkaline earth metal cations react with carbonate anions to form carbonate or bicarbonate salts.^39^ These salinities either accumulate in the A, B, or C soil horizons upon precipitation as SIC, or the groundwater carries them through underlying aquifers, and finally, these salts are accumulated in large water bodies and oceans. The solubility of carbonates at a particular soil pH would identify the long-term fate of CO_2_ sequestered through either pathway, but the atmospheric CO_2_ concentration can be effectively reduced through either mechanism of the ERW technique.^38^ From a practical aspect, ERW is deployable in a similar way to how the pH of soils can be controlled or corrected by either the addition of powdered carbonate-rich rocks to agricultural soils, or by blending such rocks with fertilizers, peat, and compost.^18^ This conventional process is known as liming owing to the conventional use of limestone and other calcium carbonate-based materials.^40^ Liming in the agricultural context of soil pH neutralization has been shown to be a net-positive CO_2_ emission practice,^41^^,^^42^ hence the use of silicates rather than carbonates is needed to achieve the negative emissions goal of ERW. Forested and urban soils can similarly be used to sequester atmospheric CO_2_ concentrations for the long term.^43^^,^^44^

Charcoal, humus, microorganisms, and decomposed plants and animal residues make up the SOC pool, whereas primary carbonates such as calcite (CaCO_3_) and secondary carbonates such as dolomite ((CaMg(CO_3_)_2_) are included in the SIC pool.^45^ These primary and secondary carbonates can be categorized into lithogenic and pedogenic carbonates, respectively. The detrital particles, which are derived from carbonate bedrock (particularly limestone) and formed under marine conditions, are referred to as primary or lithogenic carbonates. Pedogenic carbonates are the secondary carbonates in soils and subsoils. These are further classified into calcitic pedogenic carbonates (which are derived from pre-existing carbonates and are formed by calcite remobilization) and silicatic pedogenic carbonates (which come into existence because of silicate weathering). Calcitic carbonates do not play any role in net carbon sequestration, as they are formed from pre-existing carbonates. In contrast, the silicatic pedogenic carbonates are composed of the carbonation products of alkaline earth silicate minerals, which deliver net positive carbon sequestration.^46^

In ambient weathering, naturally occurring rocks weather slowly, from an anthropogenic point of view rather than a geological timescale. In the ERW process, alkaline silicate minerals are milled into fine powder form and exposed to the atmosphere under reaction-accelerating conditions. The milling increases the specific surface area of minerals, which accelerates the weathering process.^38^ The minerals can also be potentially pretreated for improving the weathering rate via mechanical, chemical, biological, or thermal means.^47^^,^^48^ However, such pre-treatment processes result in a drawback of high operational energy consumption.^49^^,^^50^ Certain soil and crop conditions accelerate the rate of silicate weathering and SIC accumulation, and these facts have been verified at relatively short timescales (months to years) under controlled and uncontrolled ambient settings using the fast-weathering natural mineral wollastonite.^7^^,^^17^^,^^18^

Wollastonite is considered as being fast weathering in contrast to other natural silicate minerals, as compiled by Palandri and Kharaka.^51^ For instance, its weathering rate at 25°C in the pH range of 5.1 to 7.7 (typical of soils) is 10^−8.88^mol m^−2^·s^−1^, compared to 10^−10.64^mol m^−2^·s^−1^ for forsterite (Mg_2_SiO_4_, the primary mineral of olivine), and 10^−12.72^mol m^−2^·s^−1^ for enstatite (MgSiO_3_, a common mineral in igneous rocks). That is, faster-weathering silicates have weathering rates that can potentially be (depending on other conditions) orders of magnitude faster than slower-weathering silicates. However, the naturally occurring silicate minerals that have demonstrated an enhanced rate of weathering and carbonation, such as wollastonite, are not as widely available,^14^ and their stocks could be depleted soon once enhanced weathering and the carbonation of silicate minerals is applied on a larger global scale. In addition, these sparse minerals may be found far from agricultural land, whereas the goal of net-negative emissions can only be realized once these silicates are accessible near the point of application.^52^ The long-term and global implementation of ERW thus relies on two choices: (1) The more abundant naturally occurring minerals can be used for serving the purpose; however, their weathering rates are slower; or (2) reactive silicates are synthesized and mass-produced, however, this is only feasible if net-negative emissions production is achieved. Silicate synthesis is traditionally a topic related to metallurgy,^16^ cement,^19^ and glass production.^53^ The second option has not yet been considered in ERW research and therefore this idea is novel to synthesize and investigate their performance both as carbon capture reagents and agricultural fertilizers.

Wollastonite (CaSiO_3_) is a polymorphic substance, naturally found in three forms: triclinic wollastonite, monoclinic (or para-) wollastonite, and pseudo- or (cyclo-) wollastonite.^54^ The first two are chain-structured and low-temperature polymorphs, whereas the third one belongs to the cyclo-silicate family and is a high-temperature polymorph, as silicate tetrahedrons (SiO_4_)^4-^ form a ring of three units (Si_3_O_9_)^6-^ at high temperature. The most common synthesis process includes the mixing of limestone (or other calcium sources) and quartz (or other silicon dioxide source). The mixture is then calcined at a temperature range of 1200–1400°C via a solid-state sintering process, which results in producing wollastonite.^55^^,^^56^

Åkermanite mineral is a product of contact metamorphism of dolostones and siliceous limestones, having the chemical formula Ca_2_Mg(Si_2_O_7_). It can be synthesized through various means; however, the conventional sintering method is common. In this method, ceramics are heated at very high temperatures, which reduces mechanical strength and fosters grain growth. Åkermanite powder was produced by Wu et al.^57^ through the sintering process at 1370°C for 6 h, Hou et al.^58^ synthesized åkermanite powder at 1350°C for 4 h, and Bodor et al.^16^ prepared akermanite powder by sintering the precursors for 24 h at 1300°C.

Bredigite mineral, having chemical composition Ca_7_Mg(SiO_4_)_4_, is a part of metasomatic rocks belonging to sanidinite facies. It is naturally formed under low pressure (less than 1–2 kbar) and high temperature (more than 800°C) conditions and is rarely found on Earth.^59^ Tavangarian and Emadi^60^ synthesized bredigite powders using a mechanical activation technique followed by annealing. Talc, amorphous silica, and calcium carbonate were used as initial reactants. The mechanical activation ranged between 10 to 60 h, whereas the subsequent annealing time was 1 h at a temperature of 1200°C.

Cuspidine is a fluorine-bearing sorosilicate mineral, having the chemical composition Ca_4_(Si_2_O_7_)(F,OH)_2_. The crystallization phase of cuspidine is monoclinic. Bodor et al.^16^ synthesized cuspidine by heating calcium oxide, fumed silica, and calcium fluoride at 1100°C for 2 h, under an inert atmosphere of Ar.

Merwinite is an orthosilicate mineral, having the chemical formula Ca_3_MgSi_2_O_8_. Hafezi-Ardakani et al.^61^ synthesized merwinite at 1300°C, and Bodor et al.^16^ synthesized merwinite by milling the precursors for 2 h, followed by 20 h of sintering at 1500°C.

Calcio-olivine (γ-C_2_S) and larnite (β-C_2_S) are the low- and high-temperature polymorphs of dicalcium silicate (C_2_S; Ca_2_SiO_4_). Calcio-olivine is orthorhombic whereas larnite is monoclinic. Fang et al.^62^ studied the mineral’s evolution and strength development by accelerated carbonation curing of β-C_2_S and γ-C_2_S phases. Both phases were sintered at 1450°C for 3 h. For avoiding the transformation of β-C_2_S to γ-C_2_S, to obtain larnite, the sintered samples were rapidly cooled.

## Synthesis and morphology

Negative emissions technologies target the removal of CO_2_ from the atmosphere as a way of combating global warming. ERW is a vital negative emissions technology that, applied globally, could remove gigatonnes of CO_2_ per year from the atmosphere. In ERW, silicate minerals exposed to the atmosphere trap CO_2_ via mineral carbonation as thermodynamically stable carbonates. From a fundamental and technological standpoint, the present challenge is that the most reactive naturally-occurring silicates are not abundant, and even those may weather too slowly in colder and dryer regions of the world, such as the Canadian Prairies. In order to design novel fast-weathering and nutrient-doped synthetic silicates for ERW in colder and dryer regions, a basic understanding of their structure and reactivity is necessitated at the nanoscale. Because of the complexity involved in experimentally characterizing mineral surfaces, recent theoretical and computational studies that complement experimental efforts have focused on exploring and understanding the structure-property relationships at silicate surfaces.^63^ Nonetheless, these computational and experimental studies are still in their infancy, resulting in a lack of clarity regarding the atomic-scale structure of low-index silicate surfaces and basic atomistic mechanisms and dynamics of CO_2_ interaction with various facets of silicates.^64^^,^^65^^,^^66^^,^^67^

The majority of the computational studies in the literature exploit the well-known atomistic simulation methods such as density functional theory (DFT)^68^^,^^69^ and empirical potentials-based molecular dynamics (MD)^70^ to model reactive silicate surfaces and their interaction with additive molecules. These two methods have their advantages as well as disadvantages. For instance, DFT offers basic insights into the thermodynamics of surface stability with the atomic and electronic structure of silicate surfaces and interface charge transfer with additive molecules. However, DFT is limited by the system size and simulation time since these calculations are extremely expensive because of large surface slab models that are necessitated to mimic silicates. An inherent limitation of DFT calculations is that they are performed at 0 K, which does not offer information about surface kinetics and dynamics. On the other hand, empirical potentials-based MD offers a window into kinetics but is unable to provide information at the electronic level. In addition, MD is limited in applicability because it heavily depends on the choice and availability of force fields. In general, because of the complexity involved in mimicking silicate surfaces, it appears that a concerted effort is required to successfully study the dynamics of CO_2_ interaction with silicate surfaces, which will be crucial to developing new negative emission technologies based on ERW.

**Figure 4:**
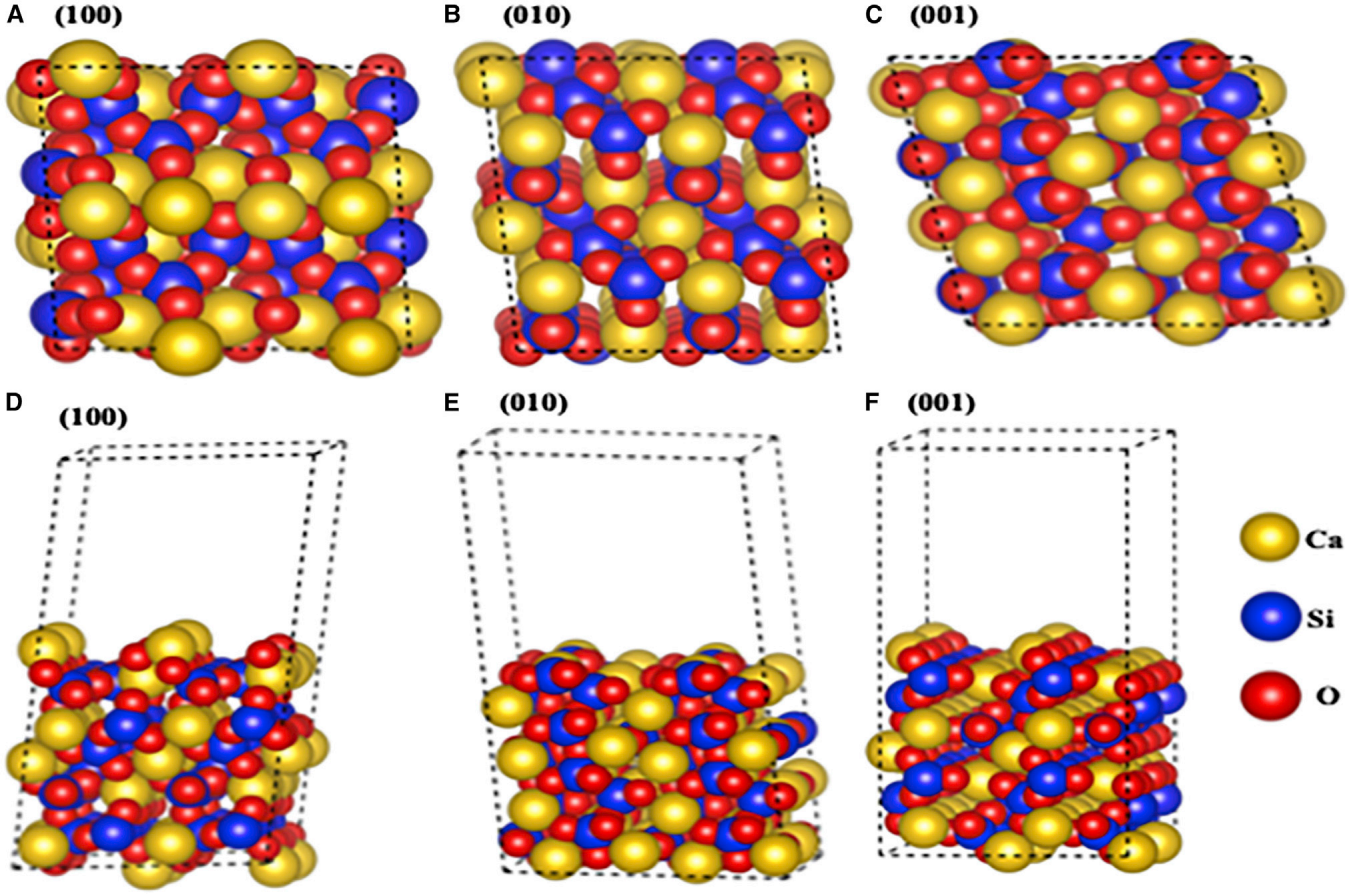
Geometrically optimized surface slabs of wollastonite crystal Normal (top) view of geometrically optimized atomic-scale structures of (a) (100), (b) (010), and (c) (001) surfaces of wollastonite depicting possible adsorption sites, and side view of (d) (100), (e) (010), and (f) (001) surfaces of wollastonite showing the intricate stacking of atomic planes. Gold, blue, and red correspond to calcium, silicon, and oxygen atoms, respectively.

Because DFT has proved to be a useful tool in predicting the structure and properties of complex materials, we recently employed DFT within the framework of the Vienna *ab initio* Simulation Package (VASP)^71^^,^^72^ to predict the atomic-scale structure low index surfaces of wollastonite (CaSiO_3_) in its most common triclinic phase using surface slab models consisting of 240 atoms.^73^ Owing to the triclinic phase, wollastonite exhibits different surface layer atomic arrangements and symmetry along different families of {100} planes. We find that the respective surface energies of (100), (010), and (001) surfaces of wollastonite are 1.349 J/m^2^, 1.103 J/m^2^, and 1.104 J/m^2^, indicating that (010) and (001) exhibit similar stabilities, whereas (100) is the least stable. Geometrically and energetically minimized atomic-scale models of (001), (010), and (100) surfaces of wollastonite are given in Figure 4. The normal and side views of the surface atomic arrangement in wollastonite evidently display the complexity associated with studying such systems. Remarkably, in each of the three surfaces, atomic layers could either constitute calcium, oxygen, or silicon, or a combination of these atoms, which further dictate the surface stability. Such intricacy is also present along the {110} and {111} families of planes. Perhaps, this is one of the potential reasons for the lack of fundamental research on low-index wollastonite surfaces. For instance, although much is known about the surface structure and properties of well-studied complex materials such as titanium dioxide,^74^ cerium oxide,^75^ strontium titanate,^76^^,^^77^ etc., the same cannot be argued about wollastonite or silicates in general. Moreover, several wollastonite low-index surfaces are Type II asymmetric according to Tasker classification,^78^ which requires careful treatment for ensuring appropriate surface cleavage so as to avoid surface energy divergence. If we move on from pure surfaces and consider surface defects, there are hardly any studies focused on their formation and stability. Because oxygen defects are ubiquitous in most materials having oxygen sublattices, they are likely to influence the surface structure and reactivity of wollastonite. In addition, dopants are often added to improve or tune the material properties, but not much is known about the role of defects and dopants in stabilizing and influencing the reactivity of wollastonite surfaces.

Based on our preliminary efforts targeting silicate surfaces and the current status of work in the field, several potential approaches could be undertaken in order to expedite and maximize the use of these technologically relevant materials. In order to design novel ERW materials at the macroscale, a fundamental understanding of their structure and reactivity is necessitated at the nanoscale. Although a macroscopic description of ERW is known, studies that uncover basic mechanisms and kinetics at the nanoscale are necessary to identify materials for ERW. Future studies illuminating the thermodynamic preference of various silicate facets that exhibit enhanced reactivity would be welcome. Atomistic-level DFT calculations that include quantum effects will be suitable to study thermodynamics and interface charge transfer in silicate-adsorbate systems. Although empirical potential-based MD is expected to offer reasonable trends, *ab initio* Molecular Dynamics (AIMD)^79^ will be more appropriate because of the complexity involved in investigating the reaction kinetics at the interface between silicate minerals and soil pore water solution. Studies focused on unraveling the influence of defects and dopants in altering the reaction dynamics at interfaces of minerals and solid solutions would be valuable. Ultimately, studies addressing the fundamental aspects of CO_2_ adsorption on silicate surfaces would be timely. For instance, insights pertaining to whether CO_2_ prefers chemisorption or physisorption and if certain facets prefer molecular versus dissociative adsorption are expected to shed light on the atomic scale mechanisms of CO_2_ capture in these minerals. Additional efforts that investigate surface coverage-dependent mechanisms for CO_2_ adsorption on silicate surfaces would aid in estimating the total amount of CO_2_ adsorbed. Overall, the aforementioned fundamental studies in tandem with experimental efforts will be imperative to guide the large-scale synthesis of novel silicates for ERW. As mentioned earlier, a co-author of the present study recently conducted first-principles modeling of CO_2_ adsorption on wollastonite surfaces for ERW application.^73^ The study used DFT to investigate the thermodynamic behavior of CO_2_ adsorption on wollastonite surfaces. The study found that the bent CO_2_ geometry bridging between the calcium atoms of wollastonite is the most favorable location for CO_2_ sequestration and indicated the importance of this binding site and the likelihood of this geometry while designing the reactive silicates for ERW application to capture and sequester CO_2_.

## Environmental and economic considerations

Synthetic fertilizer is a critical component of modern and climate-smart agriculture.^80^ However, the production of synthetic fertilizer is not without environmental and economic costs.^81^ Currently, approximately 14.6 million metric tons of phosphorus fertilizers as P are estimated to be consumed annually, in order to produce adequate food, fuel, and fiber supplies for an increasing global population.^82^

Recently there has been a push to consider the circularity of nutrients in the food-energy-water nexus. One result of this has been the deployment of struvite (NH_4_MgPO_4_·6H_2_O) as a slow-release fertilizer, recovered from human and animal wastes. Using recovered struvite has the potential to reduce the quantity of nitrogen and phosphorus discharged to the environment in areas in which it will cause damage, such as eutrophication of receiving waters, and the corresponding impacts of eutrophication such as fish kills, etc.^81^ Tonini et al.^83^ found that societal costs for recovering phosphorus from sewage sludge were 81% lower than for rock-derived phosphorus. Previous work has focused on the environmental impact of the recovery of these materials for wastewater.^84^^,^^85^ Suggesting that alternatives to conventional phosphorus fertilizers are a critical component for future sustainable agriculture.

Reactive silicates have the potential to be utilized as agricultural amendments while also enriching the soil with added nitrogen and phosphorus. These slow-weathering materials can add the necessary nutrients to the soil, and because of their inherently longer weathering spans, release them continuously over a long period of time. This is particularly relevant as it reduces the potential for nutrient runoff.^15^ quantified the potential environmental costs and benefits of using enhanced weathering for potential soil carbonation in Brazil and found a net benefit to using basalt rock for enhanced weathering. Their benefits were lower than some other studies, as they considered environmental costs, such as transportation which offset some of the anticipated benefits, which is critical to include from a holistic environmental impact perspective.

Multiple current challenges exist with respect to understanding the environmental and economic impacts of enhanced rock weather on soil health and CO_2_ sequestration. The first is accounting for all of the environmental impacts and economic implications. Tools such as life cycle assessment (LCA) and techno-economic assessment (TEA) have the potential to frame these issues systematically. The second is scale. Assuming that economies of scale will apply, the synthesis process will be less environmentally and economically intensive as production operations increase in size. A third is the regionality of application for ERW, in that it may achieve greater benefits in some geographic areas when compared to others.

**Figure 5:**
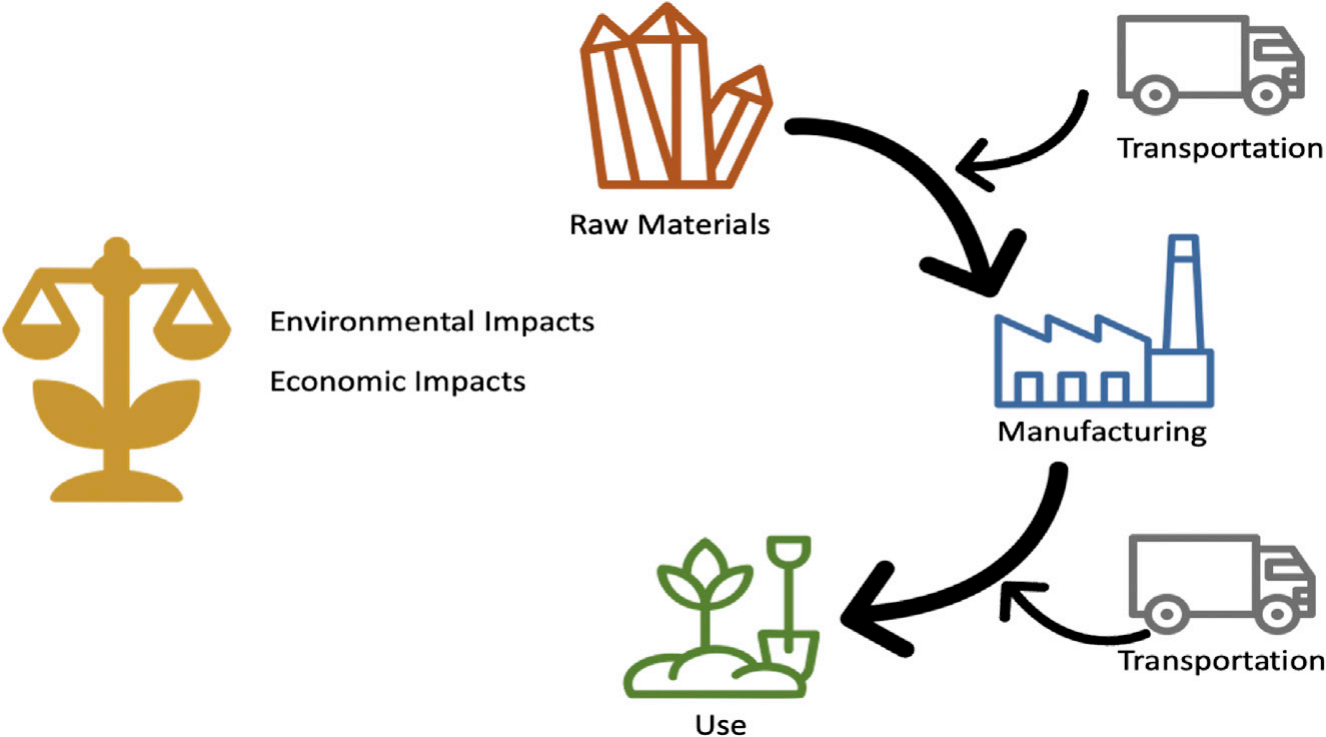
LCA stages adapted to fast-weathering silicates synthesis for fertilizer replacement

Life cycle assessment (LCA) is a systematic tool for determining the environmental impacts of a product or process across its lifetime and for the evaluation of tradeoffs. It allows for the comparison of environmental impacts across all stages of a product or process’s lifetime, to prevent burden-shifting among stages (Figure 5). To assess the true potential benefits of slow-weathering silicates for agricultural purposes, a consistent set of scopes, boundaries and functional units need to be applied across studies. In addition, if the purpose of the silicates is to replace conventional fertilizer products, their function must be compared to the function of conventional fertilizer products. Transportation is also relevant to consider, particularly when products are moved over long distances, or compared to traditional agricultural fertilizer products such as manure.^86^ Utilizing a standardized framework for comparing silicates that not only enrich the carbon content of soil through slow weathering but also have the potential to replace conventional fertilizers is critical to understanding the environmental and economic impacts and implications.

## Conclusions

The proposed approach to ERW aims at synthesizing silicate minerals with a high weathering rate to be suited for use in alkaline soils of the colder and drier agricultural lands of Western Canada and the Western United States. The following is a summary of the main investigations/future directions discussed in the present work:

• Investigating the doping of these minerals with plant nutrients for improved fertilizing ability.
• Determining the weathering mechanisms at the atomic scale and rates under normal and accelerated weathering conditions.
• Studying the role of mechanical activation and degree of crystallinity in improving their weathering performance.
• Testing the synthesized minerals for altering the agricultural soils and gauging their benefits in terms of CO_2_ capture and agriculture fertilizing capability.
• After a successful performance, conceptualizing an integrated industrial process for manufacturing these silicates at a large scale in a net-negative carbon emissions pathway.

### Limitations of the study

The cost of the synthesized minerals via doping of nutrient fertilizers could become one of the major challenges with this approach. It is, therefore, important to conduct a techno-economic analysis of the manufacturing process, which can be optimized by utilizing the waste process heat from industrial units. This information will also help in conducting a comparative analysis with other carbon capture, utilization and storage (CCUS) solutions currently present in the market, to better tackle the global warming problem.
